# Emergence of clustered synapses during the development of a nervous system

**DOI:** 10.1186/s12915-026-02539-1

**Published:** 2026-02-05

**Authors:** Yuval Balshayi, Eduard Bokman, Alon Zaslaver

**Affiliations:** Department of Genetics, Silberman Institute of Life Sciences, Edmond J. Safra Campus, The Hebrew University of Jerusalem, Jerusalem, Israel

**Keywords:** *C. elegans*, Synapse clustering, Synapse, Connectome, Neural network

## Abstract

**Background:**

Synaptic organization is central for proper transmission of neural information. Studies in invertebrates and mammalian cortices show that synapses are clustered along neurite extensions, an organization that promotes key functional roles.

**Results:**

Here we studied how these synaptic clusters emerge during the development of a nervous system. Leveraging the available *C. elegans* connectomes that span all larval developmental stages, we show that clustered synapses are formed at the early stages of the neural network development and that their occurrence further increases throughout development. These synaptic clusters significantly constitute small neural circuits that endow the network with important functional roles, such as feedback between mutually synapsing neurons and information transfer in mutually regulated neurons. Moreover, clustered synapses emerge early on during the development of the head motor system, where they facilitate the crucial 3D head swings. Finally, the synaptic clusters within these key neural circuits are maintained throughout all developmental stages and are robustly found across different individuals, further accentuating their central functional roles in neural networks.

**Conclusions:**

Clustered synaptic structures emerge early on during the development of the neural network and are consistently observed among individuals. They appear significantly in motor circuits, possibly contributing to their function.

**Supplementary Information:**

The online version contains supplementary material available at 10.1186/s12915-026-02539-1.

## Background

Neurons form polarized cell structures, known as axons and dendrites, where axonal extensions from one neuron signal dendrites of downstream neurons. In mammalian cortices, these axon-to-dendrite connections form between the arborized axons of presynaptic neurons and multiple postsynaptic neurons, and each postsynaptic neuron may receive and integrate signals from multiple axons via its own tree-like dendritic structure [1].

Contemporary technologies, which extract connectome-wide structures with fine synaptic resolution, indicate that the spatial organization of synapses is not random. Instead, postsynaptic contacts exhibit a clustered organization [2–5]. For example, in the rat barrel cortex, the vast majority of overlapping axons and dendrites are not connected, and the fraction that is connected forms synaptic clusters by connecting along the same dendritic branch [4–6]. This clustered synaptic organization is presumably instrumental for signal integration and for accurate neural firing outputs [7–10]. Furthermore, it is thought that such clusters play important roles in developmental and experience-dependent plasticity [11–14].

Interestingly, a clustered synaptic organization was also observed in the simple nervous system of *C. elegans* worms [2]. Comprising 302 neurons, connected via ~8000 chemical synapses and ~1000 gap junctions, the *C. elegans* neural network is the first fully mapped connectome for which the positions of all chemical connections are available [15–18]. In particular, this clustered synaptic organization was demonstrated within the *C. elegans* neuropil, known as the nerve ring, where most head neurons synapse with one another in a densely packed manner [15, 19, 20]. In contrast to the elaborate tree-like structures of cortical neurons, the vast majority of *C. elegans* neurons are simply structured, comprising uni- or bipolar neurites. These neurites are bundled within the nerve ring, and the clustered synaptic contacts are formed along these bundles.

The compact size of the *C. elegans* neural network and the simple structure of the neurons may limit the computational capacity of the network. To overcome this limited computational power, it has been suggested that the clustered synaptic organization supports local compartmentalized computations where distinct computations may be performed in parallel along a single neurite [2]. Indeed, functional analysis of neural activity revealed neurons in which calcium dynamics were observed in local compartmentalized regions within neurites [21–25]. For example, the RIA-type interneurons control the animals’ head bending, a result of compartmentalized and reciprocal activity between the dorsal and ventral parts of its neurite [22, 23, 25, 26]. In the RIS neuron, local activity in one neurite branch induces locomotion stop, while co-activation together with another axonal branch promotes reversal [21].

Emergence of clustered synapses in a simple invertebrate nervous system as well as in mammalian brains highlights their importance to network functions. But how does the clustered organization evolve during development and maturation of the nervous system? Do such clusters form early on during development, or do they gradually emerge as new synapses are added to the network, presumably next to pre-formed synapses? Moreover, if clustered synapses endow neural circuits with crucial functions, how stereotypic is this organization when comparing isogenic individuals?

To address these fundamental questions, we leveraged the compiled *C. elegans* connectomes spanning all developmental larval stages as well as three adult-stage connectomes [15–18]. Our analyses reveal that clustered synapses are formed early on during development and that their fraction out of the total synapses grows throughout development. They significantly constitute neural circuits that facilitate important functional roles such as feedback, integration, and motor outputs. Finally, we show that synaptic clusters are preserved throughout development and across individuals, further highlighting their potential functional roles in neural networks.

## Results

### Clustered synapses are formed early on and their fraction continues to grow throughout development

To study how synaptic clusters emerge throughout the development of a nervous system, we analyzed the nine available connectomes that span the different developmental stages of *C. elegans* [15–17]. These data comprise fully mapped connectomes of the nerve ring in four L1-stage animals (0, 5, 8, and 16 h post-hatching), one each of L2- and L3-stage animals (23 and 27 h post-hatch, respectively), and three adult animals (50 h post-hatch).

**Fig. 1:**
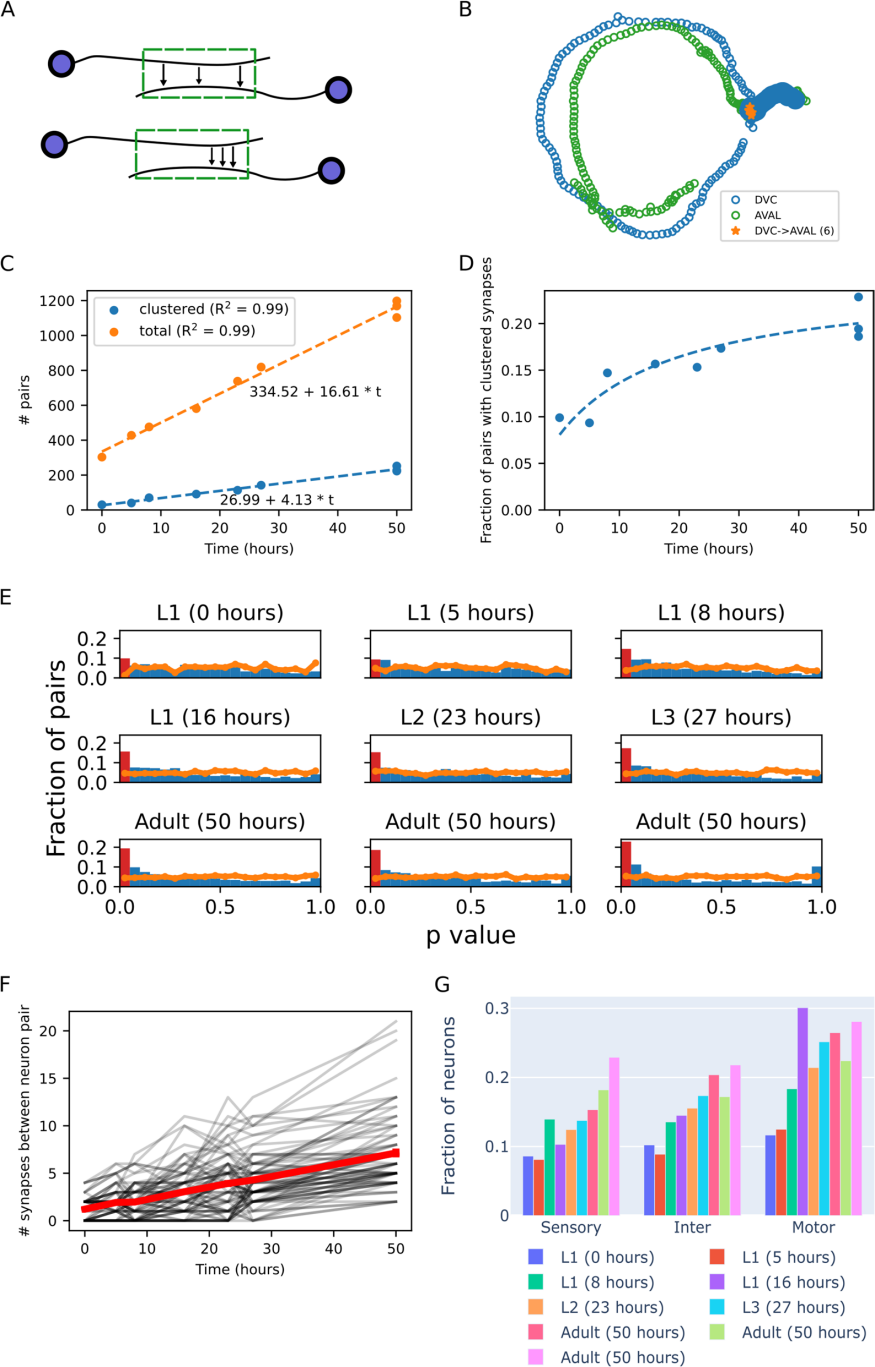
Clustered synapses are formed early during development and their fraction continues to grow throughout development. **A** Synapses may be randomly distributed (top) or clustered in a specific region (bottom) along proximal neurite regions, regions that are sufficiently close to form a synapse (green box). **B** An example of clustered synapses (orange, *p* < 0.001) along the proximal neurite region (filled blue points) between the presynaptic DVC neuron and the postsynaptic AVAL neuron in a young adult animal (Dataset 8 in [17]). **C** Number of connected pairs of neurons. Orange: total number of connected pairs. Blue: number of connected pairs in which the synapses are clustered. **D** Fraction of connected pairs of neurons with clustered synapses out of the total number of connected pairs (trend line is calculated from the linear fits in **C**). **E** Histogram of permutation test *p*-values (blue bars, see Methods). The bar representing the *p*-values which are considered significant is highlighted in red. For reference, the orange line shows the *p*-value distribution of the null model where clustering of randomly positioned synapses is tested. Real synapse positions show significantly enriched clustering compared to the null model (Kolmogorov–Smirnov test, *p* < 0.04 for all datasets). **F** Number of synapses throughout development between pairs of neurons which are connected by clustered synapses at the young adult stages. The red line shows the mean number of synapses of all pairs and the black background lines show the number of synapses for each specific pair (total 75 pairs). **G** Fraction of connected neural pairs with clustered synapses out of the total number of connected pairs of the same neuron type grouped according to the functional layer in the network of the presynaptic neuron.

We began by analyzing the simplest synaptic cluster organization in which a single presynaptic neuron synapses onto a postsynaptic neuron (Fig. 1A). We defined synapses to be clustered if the distance between the actual positions of the synapses along the neurites is significantly shorter than the distance of a random set of synaptic positions. Importantly, the position of the randomly placed synapses is drawn from proximal neurite regions, regions that are sufficiently close to one another and in which functional synapses could, in theory, be formed [2] (see Methods). For example, in the young adult animal, the DVC and AVAL neurons share a proximal stretch of neurites (Fig. 1B). Synapses between these neurons could be randomly distributed along this proximal stretch, and yet, they are significantly clustered (*p* < 0.001, permutation test) within a confined region. Notably, our findings are insensitive to the specific proximity threshold that we used (Additional file 1: Fig. S1).

During development, the total number of neural pairs connected via a synapse grows linearly, as does the number of neural pairs connected via clustered synapses (Fig. 1C). Due to the different rates, the fraction of neuron pairs connected by clustered synapses increases quickly from ~10% at the early L1 stages and saturates at the later developmental stages at ~25% (Fig. 1D). Notably, the fraction of neuron pairs connected by clustered synapses is significantly higher than the fraction expected under random distribution of synapses at all developmental stages (Fig. 1E).

Synaptic clusters may arise by adding new synapses to pre-formed synapses, or alternatively, by pruning pre-formed synapses such that only nearby clustered synapses remain. To resolve between these two possibilities, we considered the clustered synapses between pairs of neurons found in both young adult animals (originating from the same experimental cohort [17]). Next, we compared the number of synapses between these pairs of neurons throughout development. We found that the number of synapses between these pairs of neurons increases throughout development (Fig. 1F), and that the number of synapses between these pairs of neurons is greater in the young adult stage than in any of the larval stages (Additional file 1: Fig. S2). This suggests that clustered synapses form by preferential addition of synapses near pre-existing synapses.

We next asked whether neurons belonging to specific layers of the network are more likely to form clustered synapses. For this, we considered the three main layers of the network, sensory, inter-, and motor neuron layers, and assigned individual neurons accordingly. The highest fraction of clustered synapses is found in connections where the presynaptic neuron is a motor neuron (Fig. 1G). Notably, most of the postsynaptic partners of motor neurons, connected by synaptic clusters, are other neurons rather than the muscles (Additional file 1: Fig. S3). Throughout development, the fraction of the clustered neurons across all layers grows similarly.

### Mutually connected neurons form clustered synapses that appear at later developmental stages

**Fig. 2:**
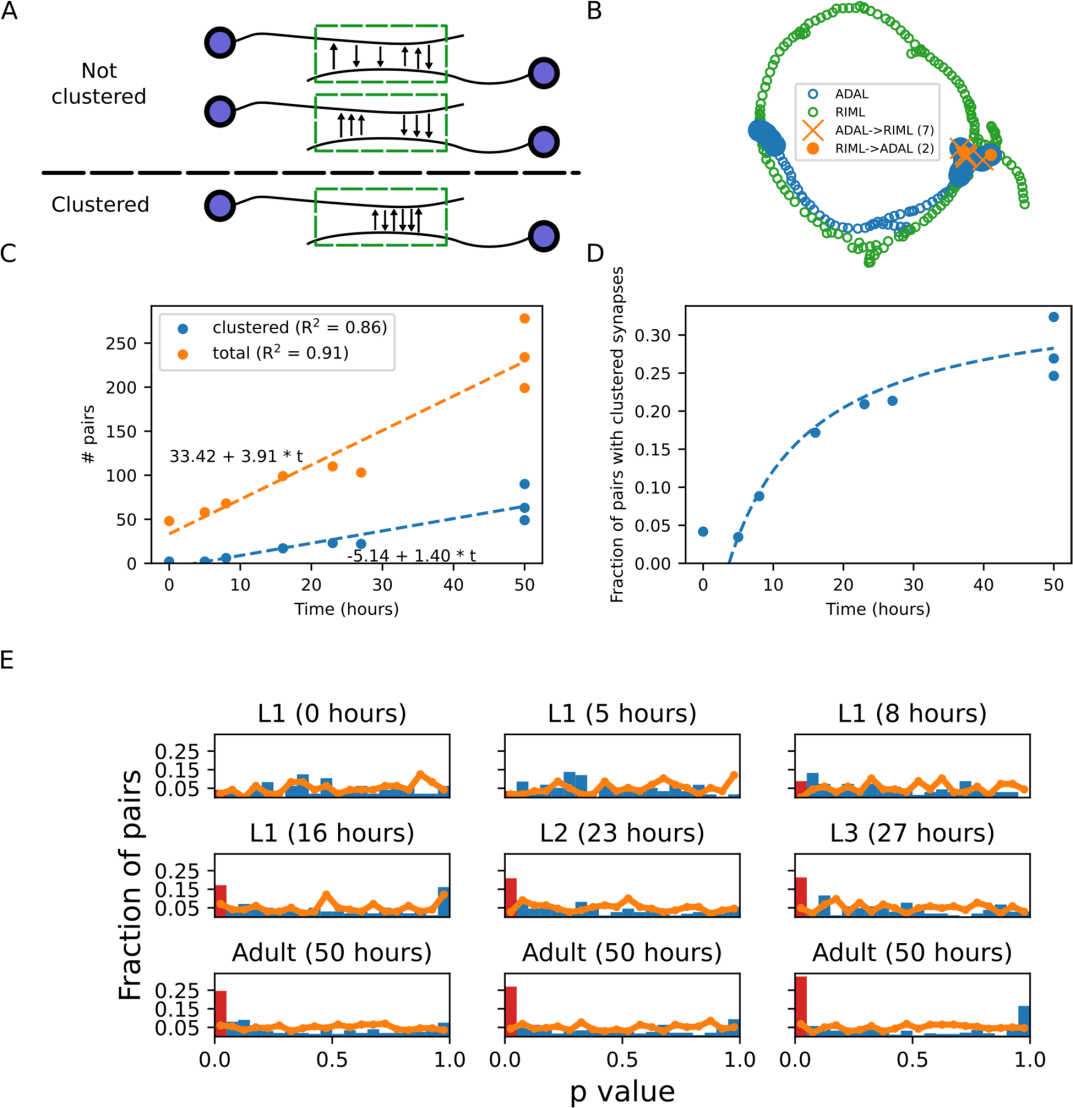
Clustered synapses between mutually connected neurons appearat later developmental stages.** A** Illustration of possible synaptic organization between mutually synapsing neurons. Top: synapses are randomly distributed along proximal regions of the neurites. Middle: incoming and outgoing synapses are segregated. While each forms a cluster, they are not clustered together for the purpose of the analysis of mutually connected neurons. Bottom: Incoming and outgoing synapses are clustered together along the proximal region of the neurite. **B** An example of mutually synapsing neurons sharing clustered synapses. Blue and green circles denote the neurite skeletons of ADAL and RIML, respectively. Filled blue circles show their proximal neurite regions. Orange X: position of the synapses from ADAL to RIML. Orange circle: position of the synapses from RIML to ADAL. Data from a young adult animal (Dataset 7 in [17]). **C** Total number of mutually synapsing neurons. Blue: number of mutually synapsing neurons in which the synapses are clustered. **D** Fraction of mutually synapsing neurons showing clustered synaptic layout out of the total number of mutually synapsing neurons. **E** Histogram of permutation test *p*-values when shuffling the positions of the synapses between the mutually synapsing neurons (blue bars, see Methods). The bar representing the *p*-values which are considered significant is highlighted in red. For reference, the orange line shows the *p*-value distribution of the null model where clustering of randomly positioned synapses is tested. Real synapse positions show significantly enriched clustering compared to the null model (Kolmogorov–Smirnov test, *p* < 0.04 for all datasets except for 0 and 16 h post-hatching).

Mutually connected neurons are small building blocks that endow neural networks with important functional roles (Fig. 2A). These feedback circuits may serve as amplifiers or short-term memories in the case of positive feedback, and oscillators in the case of negative feedback [27–30]. As these functional roles may be implemented by clustered synapses in a local compartmentalized manner [2], we next studied how such clusters emerge between mutually connected neurons throughout development (Fig. 2A, B).

In general, both the total number of mutually connected neurons as well as the number of mutually connected neurons that share clustered synapses grow linearly during development (Fig. 2C). However, the fraction of connected pairs with clustered synapses increases significantly from ~5% at the early L1 stage to ~25% at the adult stage (Fig. 2D). Moreover, while the fraction of mutually connected neurons with clustered synapses is not significantly enriched in the connectome at the L1 stage, this fraction grows quickly to become significantly enriched from the L2 stage onward (Fig. 2E). These findings suggest that clustered synapses between mutually-synapsing neurons may endow the network with pivotal functional roles, such as amplifiers or short-term memories.

### Tri-neuron circuits with clustered synapses form early during development

**Fig. 3:**
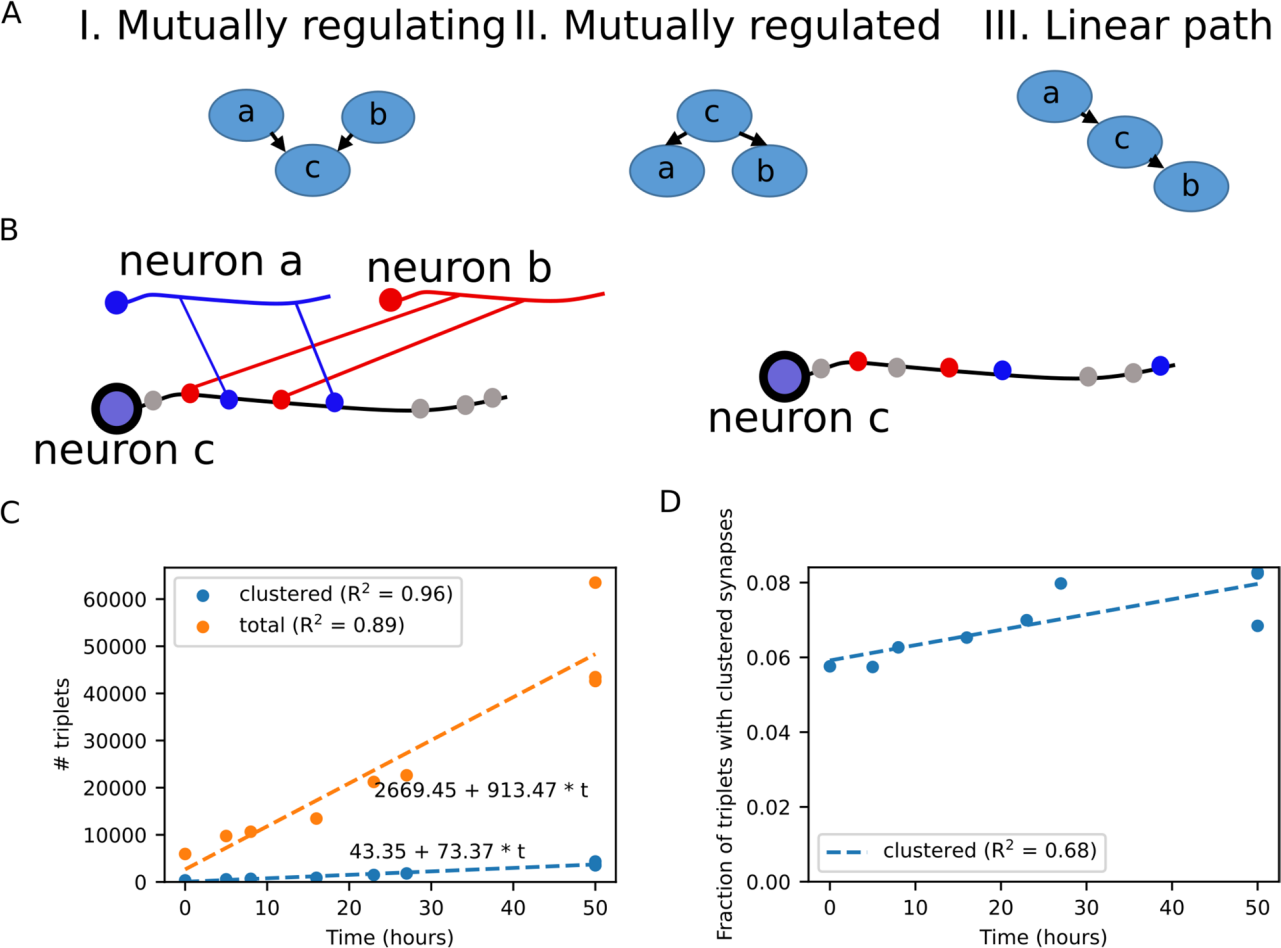
Emergence of clustered synapses in circuits consisting of three neurons.** A** Layouts of simple three-neuron circuits in which local compartmentalized activity may be performed on the neurite of the shared neuron: (I) Mutually regulating circuit—a shared neuron {c} is postsynaptic to the two presynaptic neurons {a} and {b}; (II) Mutually regulated circuit—a shared presynaptic neuron {c} synapses onto two postsynaptic neurons {a} and {b}; (III) A linear circuit—the shared neuron {c} is postsynaptic to neuron {a} and presynaptic to neuron {b}. **B** Illustration of a tri-neuron circuit and the analysis of the clustered synaptic organization on the mutual neuron {c}. We compared the mean of the distances between the actual registered synapses of neurons {a} and {b} along the neurite of the common neuron {c} (left panel) to the distances between the synaptic positions of one neuron (neuron {b} marked with red synapses, in this case) and randomly selected synaptic positions along the proximal regions on neurite {c} (right panel). **C** Number of tri-neuron circuits. Orange: total number of circuits. Blue: number of circuits in which the synapses are clustered. **D** Fraction of tri-neuron circuits showing clustered synaptic layout out of the total number of tri-neuron circuits

We next studied how synaptic clusters develop within neural circuits made of three neurons. We focused on simple circuits that lack feedback, in which two neurons connect with a third neuron as either pre- or postsynaptic neurons and regardless of other interconnections (Fig. 3A). These circuits can be classified as follows: (I) two neurons that synapse onto a third shared postsynaptic neuron, forming an input function known as mutually regulating neurons; (II) a single neuron that regulates two downstream postsynaptic neurons, a layout known as mutually regulated neurons; (III) a linear path of information flow in which one neuron synapses a second neuron, which in turn synapses a third neuron. In all these layouts, the synapses connecting the neurons may be clustered along the neurite of the shared neuron, and by this, support local compartmentalized activity (Fig. 3B). The number of such tri-neuron circuits that share clustered synapses mildly grows when compared to the total number of such tri-neuron circuits (Fig. 3C). In fact, the fraction of these tri-neuron circuits with clustered synapses grows from just 6% at the L1 stage to 8% at the adult stage (Fig. 3D).

**Fig. 4:**
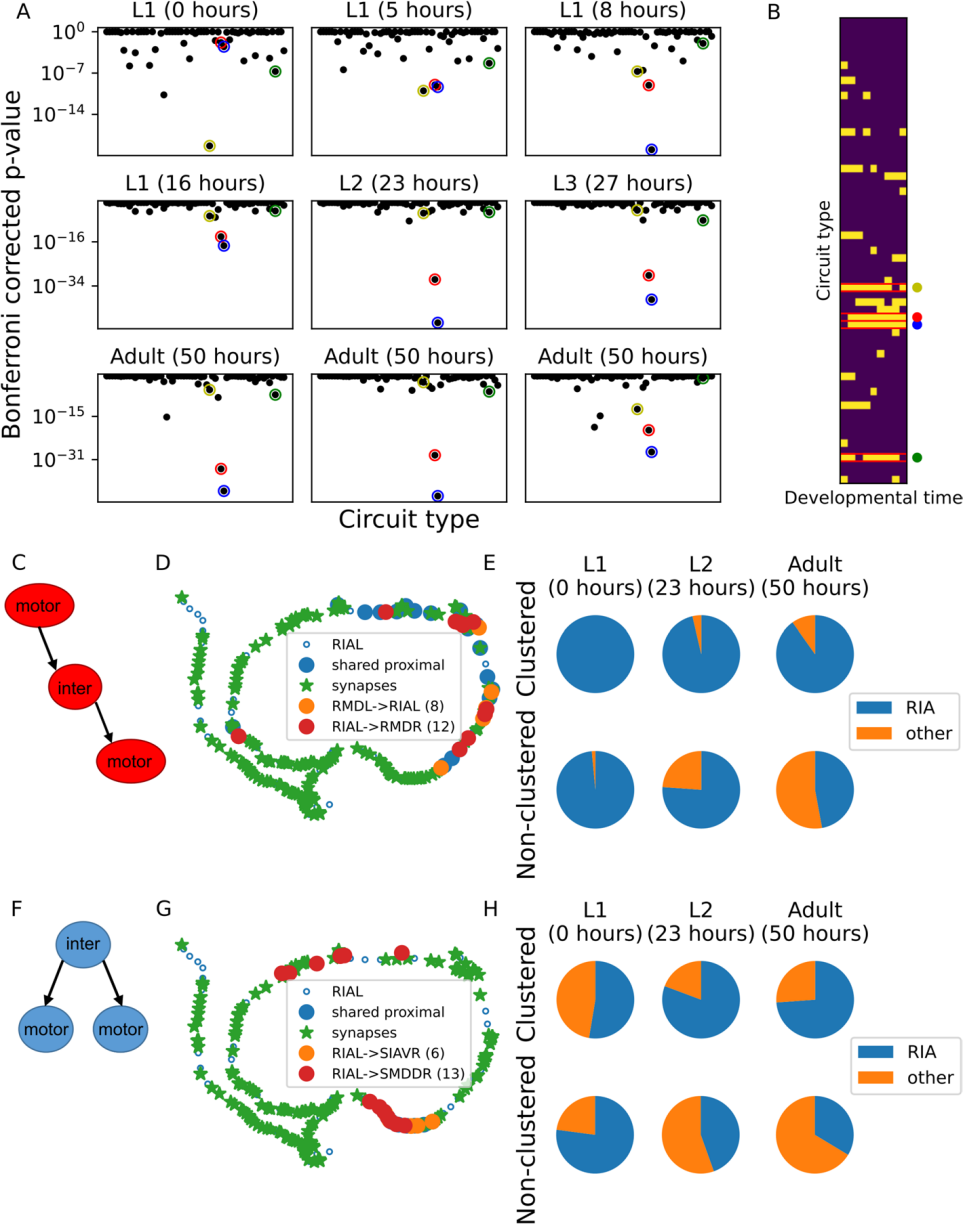
Overrepresented tri-neuron circuits are enriched with the RIA interneurons sharing clustered synapses with motor neurons.** A** Significance of the occurrence of the 63 different tri-neuron circuits with clustered synapses comprising sensory, inter-, and motor neurons (see Methods). **B** Heat map showing which circuit types are significantly clustered (yellow) in each dataset (hypergeometric test). Four circuit types that stood out are marked by colored circles: (Yellow) a presynaptic interneuron which connects to two postsynaptic interneurons; (Red) an interneuron which is both pre- and postsynaptic to motor neurons; (Blue) a presynaptic interneuron which connects to two postsynaptic motor neurons; (Green) a motor neuron which is presynaptic to both an interneuron and a motor neuron. **C** A schematic view of a linear transmission circuit in which an interneuron is both pre- and postsynaptic to two motor neurons. **D** An example of a linear transmission circuit with clustered synapses. Synapses from RMDL and to RMDR motor neurons are clustered (*p* < 0.001) along the neurite of the RIAL interneuron (taken from the L2 developmental stage, Dataset 5 in [17]). **E** Fractions of the overrepresented linear circuits with clustered (top) and non-clustered (bottom) synapses with RIA as the connecting interneuron. Shown are three representative time points along the developmental axis (see Additional file 1: Fig.S5A for analysis of all developmental stages). **F** A schematic view of a mutually regulating circuit in which an interneuron synapses onto two postsynaptic motor neurons. **G** An example of a mutually regulating circuit with clustered synapses. Synapses to the SIAVR and SMDDR motor neurons are clustered (*p* = 0.001) along the neurite of the presynaptic interneuron RIAL (taken from the L2 developmental stage, Dataset 5 in [17]). **H** Fractions of the overrepresented mutually regulating circuits with clustered (top) and non-clustered (bottom) synapses with RIA as the shared presynaptic interneuron. Shown are three representative time points along the developmental axis (see Additional file 1: Fig. S5B for analysis of all developmental stages)

We next analyzed whether the tri-neuron circuits connected via clustered synapses are associated with a specific type of the tri-neuron circuits while additionally considering the neuron layers comprising the circuit, a feature that may indicate their potential functional roles. For this, we assigned neurons to one of the network layers: sensory, inter-, and motor neuron layers. Overall, there are 63 different configurations to generate the three circuit layouts while considering the three neuron types. Notably, of the 63 possible configurations, only four circuits were overrepresented within the network throughout most developmental stages (Fig. 4A, B, Additional file 1: Fig. S4A).

Interestingly, a detailed analysis revealed that the RIA interneuron type is significantly overrepresented in two of these circuits throughout all developmental stages (except for the very early L1 stage; Additional file 1: Fig. S5A-B). The first circuit depicts a linear layout in which the interneuron is pre- and postsynaptic to motor neurons (Fig. 4C, D). The vast majority of these circuits, which also form clustered synapses, consist of RIA as the interneuron throughout all developmental stages (Fig. 4E). In contrast, in non-clustered synapses, the fraction of RIA drops during development to be half of all interneurons at the adult stage (Fig. 4E and Additional file 1: Fig. S5A). In the second circuit, the RIA interneuron is presynaptic to two motor neurons (Fig. 4F, G). Of the circuits with clustered synapses, the fraction of RIA is ~half at the L1 stage and grows to ~75% at the adult stage (Fig. 4H and Additional file 1: Fig. S5B). In contrast, the fraction of RIA in circuits with non-clustered synapses is ~75% at the early L1 stage and drops to ~30% at the adult stage.

These findings indicate that only a few tri-neuron circuits are overrepresented having clustered synapses throughout most developmental stages. Moreover, the pair RIA interneurons are particularly enriched within two of the circuits where they either receive or transmit information to motor neurons. Indeed, the RIA neurons are central in regulating head movement via compartmentalized activity across different neural modules [18, 20, 22, 23, 26]. The significant overrepresentation of clustered synapses in RIA, throughout all developmental stages, further shows the compartmentalization at the synaptic level.

### Clustered synapses are conserved in the developing nervous system

The above analyses showed that clustered synapses are formed early on during development and that they appear preferentially in specific circuits. But how variable are the identities of the clustered synapses between individuals and how well preserved are they across the different developmental stages?

**Fig. 5:**
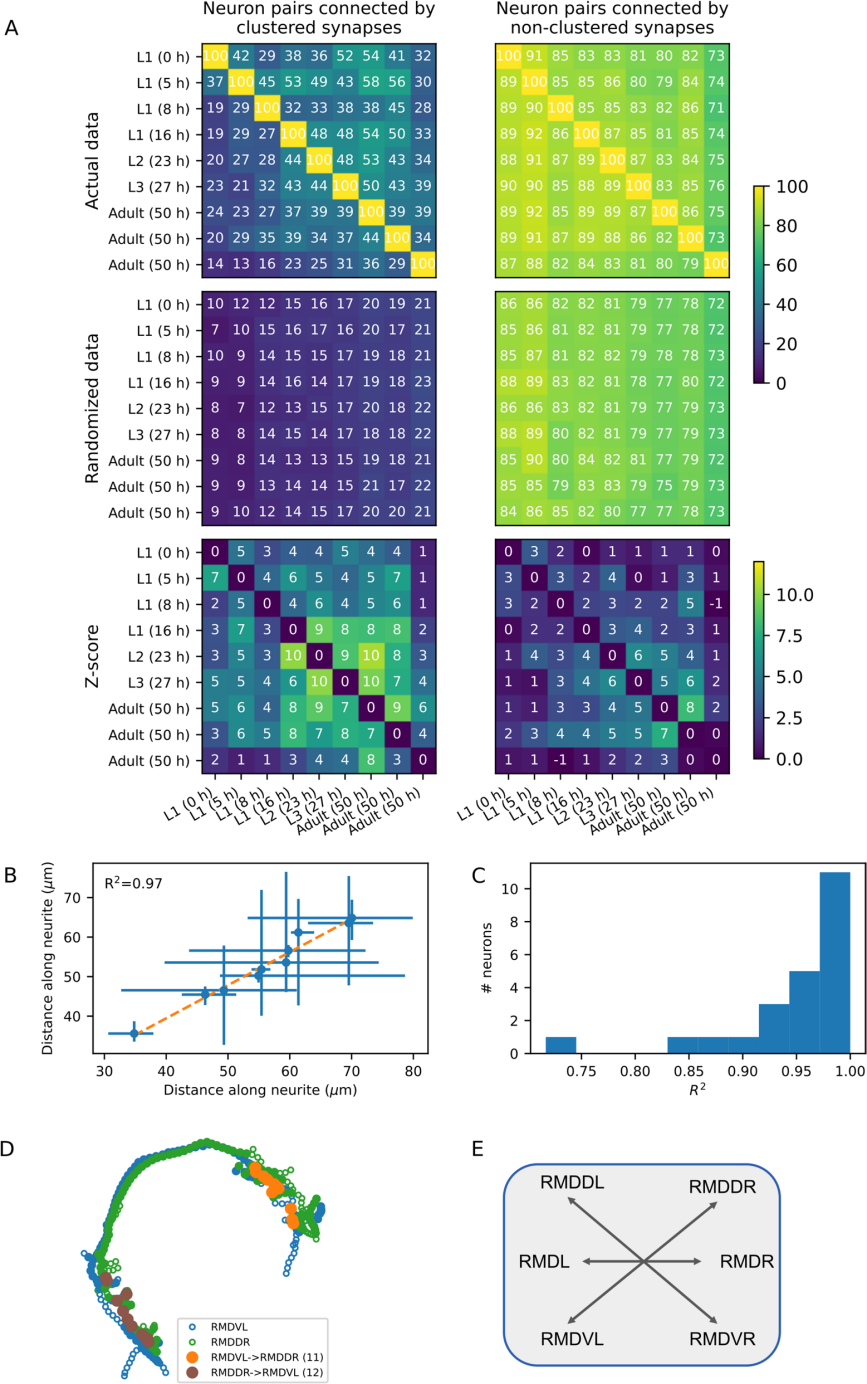
Clustered synapses are significantly preserved throughout development and across individuals.** A** Top: A pairwise analysis indicating the % of neural pairs with clustered synapses in one developmental stage (denoted by the row) that also have clustered synapses in the other developmental stage (denoted by the column). Middle: Repeating the analysis using bootstrap to correct for the number of neural pairs with clustered and non-clustered synapses (see Methods). *N* = 50 iterations. Bottom: *z*-scores calculated based on the top and the middle matrices. Left and right panels denote the neural pairs connected by clustered and non-clustered synapses, respectively. The values of the rightmost and the bottom are for the adult animal connectome compiled by [16]. All developmental stages as well as the two other adult connectomes are from [17]. Color bars indicate percent values for the top and the middle matrices, and *z*-scores for the bottom matrices. **B** Comparison of the synaptic cluster positions along the neurite of RIAL. Each point represents the mean distance along the neurite of all the synapses between RIAL and one of the (pre/post) synapsing neurons. The error bars show the region in which synapses appear. On the *x*- and *y*-axes are the positions extracted from Dataset 7 and Dataset 8 (both young adult connectomes from [17]), accordingly. **C** Distribution of *R*^2^ values for comparisons of synaptic cluster positions between the two young adult connectomes for different neurons. **D** The neurites between the RMD neurons form clustered synapses. Shown as an example are the synapses along the neurites of the RMDVL (blue) and the RMDDR (green) neurons. These clustered synapses are preserved throughout all developmental stages. Filled and open circles indicate proximal and non-proximal regions of the neurites, respectively. Orange: positions of synapses from the RMDVL to the RMDDR neurons. Brown: synapses in the opposite direction. **E** A schematic layout of the contralateral connectivity of the RMD neurons. These contralateral neurons share clustered synapses throughout all developmental stages

To address these questions, we performed a pairwise analysis across all developmental stages and calculated the % of shared neural pairs with clustered synapses (out of the total pairs with clustered synapses between each two developmental stages (Fig. 5A topleft, and see Methods for details). The mean percent of shared clustered synapses is 35.9 ± 10.7% when comparing across all developmental stages, and rises to 39.5 ± 7.1% when comparing between sequential developmental stages. Furthermore, comparing connectomes of only adults reveals a similar ~40% preservation degree of clustered synapses. When repeating this analysis for neural pairs connected via non-clustered synapses, a higher fraction (84.5 ± 4.9%) of shared neural pairs is obtained (Fig. 5 topright). However, neural pairs connected via non-clustered synapses constitute the majority of connected neural pairs: In fact, non-clustered synapses make up 80%–90% of all neural connections across the different developmental stages, while clustered synapses constitute only 10%–20% of the total neural connections (Fig. 1D). Therefore, taking the numbers and specific identities of clustered and non-clustered connections into account, it is expected that clustered neural pairs will show a lower preservation degree (15.1 ± 4.3%, Fig. 5A middle panels, and see Methods for extended explanation). In contrast, the mean of the expected fractions of non-clustered synapses remains similar to the non-randomized pairs (~80.6 ± 4.3%). Thus, the fraction of shared clustered synapses is significantly higher than randomly expected, as also demonstrated by the *z*-score analysis, where the mean *z*-score values for clustered and the non-clustered synapses are 5.3 ± 2.4 and 2.5 ± 1.8, respectively (Fig. 5A bottom panels).

The compiled connectomes show considerable inherent variability with regard to the number of synapses registered between pairs of connected neurons [17]. Consequently, neural pairs sharing one or two synapses in one connectome may lack these synapses in another connectome, and hence, not be categorized as connected. This variability, stemming from the “low numbers” principle, may therefore bias our analysis. To control for this inherent variability, we repeated the analysis, this time considering sets of connected pairs that share at least three or four synapses. Similar higher values of shared clustered synapses were observed in these more stringent analyses, further underscoring the notion that clustered synapses are significantly more conserved than non-clustered synapses (Additional file 1: Fig.S7-S8).

Furthermore, we asked whether the position of the preserved clusters along the neurites is also maintained between individuals. For this, we compared the location on the neurites that these clusters appear in the two young adult connectomes. We found that the order and the distance of clusters are highly conserved (Fig. 5B, C). For example, the position of the clusters along the RIAL neurite is highly correlated between the two adult connectomes (*R*^2^ = 0.97, Fig. 5B). This high correlation was typical of all neurons (mean *R*^2^ = 0.95, std = 0.06, Fig. 5C). Together, the identity of the preserved clusters as well as their position along the neurites is highly conserved between isogenic individual animals. Interestingly, a single type of neuron, RMD, is consistently found to be connected via clustered synapses throughout all developmental stages (Fig. 5D and Table 1). The RMD type includes six motor neurons with left/right and dorsal/ventral symmetry. These neurons form clustered synaptic connections contralaterally at flattened protrusions [18], for example, the dorsal-right RMDDR neuron and the ventral-left RMDVL neuron (Fig. 5E). Each neuron is also connected to head muscles via neuromuscular junctions. This way, the RMD neurons control head movement in all directions, and the clustered contralateral synapses presumably allow efficient synchronization when controlling opposite side muscles [31]. Furthermore, the RIA type neurons, which are the main neurons to participate in clustered synaptic contacts (Fig. 4), also form bi-directional clustered synaptic contacts with the RMD neurons to control head movement. These bi-directional clustered synapses are also preserved throughout development from the middle L1 stage and on (Table 1). These findings demonstrate how clustered synaptic structures evolve early on during development and are preserved throughout development. Moreover, they appear invariably in all three adult animals, further demonstrating the exact positioning of synapses among individual animals.

**Table 1:** Synaptic clusters preserved throughout development. The number of synapses between pairs of neurons that share clustered synapses throughout the different developmental stages. The connections between all pairs of neurons have significant clustered synapses, except for those indicated as not significant (ns). Note the top rows consisting of the RMD neurons which are interconnected via clustered synapses throughout all developmental stages

Presynaptic neuron	Postsynaptic neuron	L1 (0 h)	L1 (5 h)	L1 (8 h)	L1 (16 h)	L2 (23 h)	L3 (27 h)	YA (50 h)	YA (50 h)
RMDDL	RMDVR	4	6	6	3	9	6	8	11
RMDDR	RMDVL	4	5	6	5	9	10	14	12
RMDL	RMDR	2	4	5	8	7	7	7	12
RMDVL	RMDDR	4	5	6	5	10	9	16	11
RMDVR	RMDDL	3 (ns)	6	5	6	10	11	14	10
AIBL	SMDDR	2 (ns)	2 (ns)	3	3	3	5	4	5
AIBR	SMDDL	2 (ns)	4	3	2	5	4	2 (ns)	6
RMDL	RIAR	2 (ns)	2 (ns)	4	3	5	4	8	8
RMDR	RIAL	2 (ns)	3 (ns)	3	3	5	6	5	7
RMDR	RMDL	2 (ns)	3	7	6	9	8	5	7 (ns)
SAADL	RIML	2	2	3 (ns)	7	3	5 (ns)	8	5
SAADL	RIMR	2	2 (ns)	2 (ns)	4	6	3	9	10
SAAVL	AVAL	0 (ns)	4	3 (ns)	5	11	10	19	19
SAAVR	AVAR	3 (ns)	5	3 (ns)	4	13	10	26	21

## Discussion

Rapidly advancing technologies now make it possible to document individual synapses within intact brain regions and to compile detailed synapse-based connectomes, revealing that synapses are organized in clusters [2–5]. Synaptic clustering is presumably shared across the animal kingdom, as clustering has been observed in mice cortices as well as in the invertebrate *C. elegans* worm. This converging structural feature strongly suggests that synaptic clusters have key functional roles in neural circuits that allow signals to propagate in an accurate and timely manner [9]. Herein, leveraging the available time-series connectomes of *C. elegans* [15–17], we studied how these clustered synaptic structures emerge during the development of a neural network.

Naively, there are two possible scenarios for the emergence of clustered synaptic structures: These structures could be formed early on during development and maintained throughout development. Alternatively, at early stages, synapses are uniformly dispersed between connected neurons, and clustered synapses could emerge throughout development via one of the two possible sequences: either new synapses are preferentially added next to pre-existing synapses, or remote synapses are pruned, such that only closely-positioned synapses remain in place. Our analyses show that while some crucial clusters form early on during development (*e.g.*, head movement circuitry during the L1 stage), most clusters emerge as new synapses are added next to pre-existing synapses (Fig. 1F and Additional file 1: Fig. S2).

One theory regarding synaptic contacts is known as Peters’ rule, which postulates that synaptic connections are randomly formed wherever axons pass in close proximity to a dendrite [32–34]. An alternative theory, known as Sperry’s key-and-lock mechanism, posits that synapses form between two neurons which are preprogrammed to connect via expression of specific adhesive proteins [35, 36]. Our study shows that a significant fraction of the synapses is in clusters rather than being randomly positioned along the neurite. This clustered synaptic positioning may be a result of location-specific signaling between the pre- and postsynaptic neurons which specifies where synapses should form. This may also explain the formation of new synapses next to pre-existing synapses. While our study points to a more controlled mechanism of synapse positioning that may support Sperry’s theory, it does not preclude Peters’ rule in the more global sense, which refers to the general connectivity between neuron types [34].

Analysis of small circuits composed of three neurons revealed a few layouts that stood out as significantly overrepresented throughout development to consist clustered synapses. Strikingly, the bilateral RIA interneurons appear in the vast majority of these circuits (Fig. 4). The RIA interneurons output primarily onto motor neurons, including the RMD motor neurons that control head movement [18, 22–24]. Interestingly, the RMD neurons themselves connect to their contralateral partners via clustered synapses as well as to the RIA neurons. Moreover, all these clustered synapses are formed early on during development and are preserved throughout all developmental stages (Fig. 5 and Table 1). Thus, the emergence of clustered synapses early on during development may support key motor functions that are crucial at the very first hours of the developing animal and throughout its entire life.

The mutually connected neuron pairs and the tri-neuron circuits are embedded within a dense neural net in which each neuron is part of several different circuits. Indeed, it has been shown that mutually synapsing as well other tri-neuron circuits make small building blocks within larger circuits [29, 37–39]. These larger circuits consist of interneurons that integrate sensory information and motor neurons that receive multiple inputs from interneurons to promote a synchronized coordinated movement. Clustered synaptic structures along the neurites of these inter and motor neurons may be crucial for such fine locomotion patterns.

Notably, functional studies of selected interneurons revealed calcium transients within compartmentalized regions of the neurites [21–25, 40, 41]. For example, the RIA interneurons control the animals’ head movement via compartmentalized and reciprocal activity between the dorsal and ventral parts of its neurite. Compartmentalized activity was also observed in the nerve ring segment and the axonal branch of the RIS neuron: activity in the nerve ring process alone correlates with locomotion stop, and coactivity with the axonal branch promotes reversal events. These studies further highlight the functional importance of synapse location, demonstrating how neural information can be transferred in a neurite-local manner without involving the entire neuron. Furthermore, as several compartments may function in parallel along a single neurite, the emergence of synaptic clusters can greatly enhance the computational capacity of the entire neural network. This feature may be particularly relevant in the early stages of a developing neural network, as clustered synapses in key circuits may enhance functional repertoires despite the fact that the network is not yet fully wired and many of the neural connections are yet to be established.

To this end, the *C. elegans* neuropil (nerve ring) is organized into four layered regions (strata) that segregate early on during embryogenesis [20]. Interestingly, a group of ~30 “rich club” neurons [18, 42, 43], which share multiple synaptic partners, bridge across these strata (including RIA and RIS neurons mentioned above). Synaptic clusters along the neurites of these highly connected neurons are partitioned across different strata, presumably to transduce information across these segregated layers.

Our analyses indicate that synaptic structures are found within key neural circuits, and that they are significantly preserved throughout development and across adult individuals. However, it is somewhat surprising that the preservation degree of these clustered structures across isogenic adult animals is only ~40% (Fig. 5). Even when exclusively considering synaptic partners that share more than a few synapses, thus overcoming the inherent variability associated with a small number of synapses, the preserved degree between adult animals reaches only ~50% (Additional file 1: Fig. S7–S8). Indeed, network analyses throughout all developmental stages, including the three adult-stage connectomes, demonstrated considerable differences in both the number of synapses and in the adjacency of the neurites [17, 34]. Thus, the low preservation degree in the identity of the clustered synapses may be a direct result of this high inter-individual variability. Nevertheless, our analyses indicate that in crucial circuits, the clustered structures are preserved during development and across individuals.

The majority of the *C. elegans* synapses are polyadic, that is, they have several postsynaptic neurons assigned [15]. These postsynaptic neurons were determined by proximity to the presynaptic densities; however, it is not clear how many of them are functionally connected. The polyadic structure may therefore provide further constraints on synapse locations that we did not consider in our analysis, and which may increase the number of clusters with neuronal pairs that are not functionally connected. In addition, it could be that neurons utilizing specific neurotransmitters preferentially form synapse clusters. For example, we found the motor system, characterized by cholinergic signaling, is significantly enriched with clustered synapses.

## Conclusions

This study demonstrates that clustered synaptic structures, which may provide key functions, emerge early on during the development of a neural network. This may explain why these clustered structures are ubiquitously found in diverse animal species.

## Methods

### Datasets

Datasets used in this study were retrieved and compiled from [2, 16–18]. Each dataset was compiled from a series of electron micrographs and provides coordinates of points along the skeleton of the neurons, as well as the spatial positions of the synapses (by associating the pre- and postsynaptic positions with the skeleton points). In regions in which there were large gaps between skeleton points (defined as larger than the distance between three consecutive electron micrographs), additional points were added by interpolation so that the gaps would be approximately the slice distance.

### Testing significance of clustering of synapses between a pair of neurons

We used a permutation test (Figs. 1E and 2E) to test whether synapses are clustered between a pair of pre- and postsynaptic neurons: the mean distance between the real synapse positions along the presynaptic neuron was compared to the mean distance between randomly selected positions along the region of the neurite that is sufficiently close (proximal) to form a synapse with the postsynaptic neuron.

To determine the axonal proximal regions (the regions that could potentially form a synapse), we first calculated the median distance between all pre- and postsynaptic skeleton positions in the dataset. We then defined proximal regions as points along the presynaptic neuron that are closer to a postsynaptic neuron point than the calculated median distance (as also defined in [2]). The permutation test was run 1000 times, or for all possible permutations if there were fewer possible permutations.

The synapse clustering *p*-value was defined as the fraction of tests in which the mean distance between the randomly positioned synapses was less than the mean distance between the actual synapses. This test was performed for every pair of neurons connected by at least two synapses, as well as for mutually connected neurons. Neurons connected via a single synapse (no meaning to a distance between synapses) or with fewer proximal points than synapses (no possible permutations) were excluded from the analysis.

To validate the results, we also ran the test for synapses in randomized locations. For each pair of neurons tested, we got two *p*-values, one for the clustering of the real synapses and one for the clustering of the randomized synapses. The histogram of *p*-values is presented in Figs. 1E and 2E. The blue bars represent the histogram of the real synapses while the orange line represents the randomized synapses.

### Testing clustering significance of synapses of two neurons along the neurite of a third common neuron

To determine whether the synapses of two neurons (A and B) are clustered along the neurite of a third common neuron (C), we applied the following permutation test: in each permutation, the mean distance between every pair of A and B synapses along the neurite of neuron C was compared to the mean distance between the positions of the real synapses of one of the neurons and randomly selected synapses from all synapse positions along neurite C. This test was run 1000 times while shuffling the synapses of A, and 1000 times shuffling the synapses of B. When the number of possible permutations was less than 1000, all possible permutations were considered. From each set of tests, a *p*-value was calculated as the fraction of tests in which the mean distance between the randomly positioned synapses was less than the mean distance between the real synapses. Finally, the *p*-value for clustering in the triplet was defined as the maximum between *p*-values when the synapses of A were shuffled and when the synapses of B were shuffled.

### Tightly clustered tri-neuron circuits

We performed both permutation tests and hypergeometric tests to study which specific three-neuron circuits have a higher probability of forming clustered synapses along the neurite of the common neuron. For the permutation tests, we shuffled which of the tri-neuron circuits are clustered 3000 times, and compared the number of tri-neuron circuits which were clustered after shuffling with the real number of circuits that have clustered synapses. The hypergeometric tests were performed using the total number of circuits, the total number of circuits with clustered synapses, the total number of circuits of each type, and the number of circuits of the specific type that have clustered synapses.

### Analysis of synaptic clusters preservation

We performed a pairwise comparison across all datasets. First, we compiled a list of all connected pairs of neurons that share at least two synapses (clustered and non-clustered) as well as a list of only the pairs of neurons that share clustered synapses (sharing at least two synapses) in each dataset. To calculate the fraction of neural pairs with clustered synapses in one dataset that are also clustered in another dataset, we counted the number of pairs with clustered synapses that appear in both lists of pairs with clustered synapses (one for each dataset) and divided this number by the number of neural pairs that appear both in the list of pairs with clustered synapses for one of the datasets and in the list of connected pairs for the other dataset, thus controlling for pairs that could not be analyzed in the second dataset since they were not connected (Additional file 1: Fig. S6). For the comparison of pairs with non-clustered synapses, we repeated this analysis using the complement of the list of pairs with clustered synapses in each dataset.

To calculate the expected percentages of shared pairs between datasets, we ran the above calculation 50 times, each time shuffling the identities of the clustered pairs in one of the two datasets compared. In this way we got a distribution of the number of specific pairs clustered in both datasets, under the assumption that only the number of pairs connected by clustered synapses is conserved but their identities are randomized. We then calculated the mean and standard deviation of these distributions for every two datasets and used these values to calculate the *z*-scores.

## Supplementary Information

Additional file 1. Supplementary Figures S1–S8. A pdf file consisting of eight supplementary figures. Supplementary Fig. 1. Control analysis for synaptic clusters showing that the chosen neurite proximity does not affect the cluster dynamics throughout development. Supplementary Fig. 2. The number of synapses forming clustered connections increases between larval and adult stages. Supplementary Fig. 3. Number and fraction of postsynaptic neurons and muscles which are connected by clustered synapses to a presynaptic motor neuron. Supplementary Fig. 4. Emergence of tri-neuron circuits during development. Supplementary Fig. 5. The RIA interneuron emerges as the primary interneuron within the overrepresented tri-neuron circuits to form clustered synapses. Supplementary Fig. 6. Comparison of neuron pairs with clustered synapses between datasets. Supplementary Fig. 7. Similarity of neuron pairs connected by at least three synapses. Supplementary Fig. 8. Similarity of neuron pairs connected by at least four synapses. 

## Data Availability

All data generated or analysed during this study are included in this published article, its supplementary information files and publicly available repositories at [(10.5281/zenodo.17846991).

## References

[1] Kandel ER, Koester JD, Mack SH, Siegelbaum SA. Principles of neural science, sixth edition. McGraw Hill LLC; 2021.

[2] Ruach R, Ratner N, Emmons SW, Zaslaver A. The synaptic organization in the Caenorhabditis elegans neural network suggests significant local compartmentalized computations. Proc Natl Acad Sci. 2023Jan 17;120(3):e2201699120. 36630454 10.1073/pnas.2201699120 PMC9934027

[3] Qian P, Manubens-Gil L, Jiang S, Peng H. Non-homogenous axonal bouton distribution in whole-brain single-cell neuronal networks. Cell Rep. 2024 [cited 2024 July 27];43(3). Available from: https://www.cell.com/cell-reports/abstract/S2211-1247(24)00199-2 10.1016/j.celrep.2024.113871 38451816

[4] Udvary D, Harth P, Macke JH, Hege HC, Kock CPJ de, Sakmann B, et al. The impact of neuron morphology on cortical network architecture. Cell Rep. 2022 [cited 2024 July 27];39(2). Available from: https://www.cell.com/cell-reports/abstract/S2211-1247(22)00429-6 10.1016/j.celrep.2022.110677 PMC9035680 35417720

[5] Kasthuri N, Hayworth KJ, Berger DR, Schalek RL, Conchello JA, Knowles-Barley S, et al. Saturated reconstruction of a volume of neocortex. Cell. 2015July 30;162(3):648–61. 26232230 10.1016/j.cell.2015.06.054

[6] Motta A, Berning M, Boergens KM, Staffler B, Beining M, Loomba S, et al. Dense connectomic reconstruction in layer 4 of the somatosensory cortex. Science. 2019;366(6469):eaay3134. 10.1126/science.aay3134 31649140

[7] Iascone DM, Li Y, Sümbül U, Doron M, Chen H, Andreu V, et al. Whole-neuron synaptic mapping reveals spatially precise excitatory/inhibitory balance limiting dendritic and somatic spiking. Neuron. 2020May 20;106(4):566-578.e8. 32169170 10.1016/j.neuron.2020.02.015 PMC7244395

[8] Katz Y, Menon V, Nicholson DA, Geinisman Y, Kath WL, Spruston N. Synapse distribution suggests a two-stage model of dendritic integration in CA1 pyramidal neurons. Neuron. 2009July 30;63(2):171–7. 19640476 10.1016/j.neuron.2009.06.023 PMC2921807

[9] Gidon A, Segev I. Principles governing the operation of synaptic inhibition in dendrites. Neuron. 2012July 26;75(2):330–41. 22841317 10.1016/j.neuron.2012.05.015

[10] Polsky A, Mel BW, Schiller J. Computational subunits in thin dendrites of pyramidal cells. Nat Neurosci. 2004;7(6):621–7. 15156147 10.1038/nn1253

[11] Chen JL, Villa KL, Cha JW, So PTC, Kubota Y, Nedivi E. Clustered dynamics of inhibitory synapses and dendritic spines in the adult neocortex. Neuron. 2012Apr 26;74(2):361–73. 22542188 10.1016/j.neuron.2012.02.030 PMC3340582

[12] Frank AC, Huang S, Zhou M, Gdalyahu A, Kastellakis G, Silva TK, et al. Hotspots of dendritic spine turnover facilitate clustered spine addition and learning and memory. Nat Commun. 2018Jan 29;9(1):422. 29379017 10.1038/s41467-017-02751-2 PMC5789055

[13] Kleindienst T, Winnubst J, Roth-Alpermann C, Bonhoeffer T, Lohmann C. Activity-dependent clustering of functional synaptic inputs on developing hippocampal dendrites. Neuron. 2011Dec 22;72(6):1012–24. 22196336 10.1016/j.neuron.2011.10.015

[14] Makino H, Malinow R. Compartmentalized versus global synaptic plasticity on dendrites controlled by experience. Neuron. 2011Dec 22;72(6):1001–11. 22196335 10.1016/j.neuron.2011.09.036 PMC3310180

[15] White JG, Southgate E, Thomson JN, Brenner S. The structure of the nervous system of the nematode Caenorhabditis elegans. Philos Trans R Soc Lond B Biol Sci. 1986Nov 12;314(1165):1–340. 22462104 10.1098/rstb.1986.0056

[16] Cook SJ, Jarrell TA, Brittin CA, Wang Y, Bloniarz AE, Yakovlev MA, et al. Whole-animal connectomes of both *Caenorhabditis elegans* sexes. Nature. 2019;571(7763):63–71. 31270481 10.1038/s41586-019-1352-7 PMC6889226

[17] Witvliet D, Mulcahy B, Mitchell JK, Meirovitch Y, Berger DR, Wu Y, et al. Connectomes across development reveal principles of brain maturation. Nature. 2021;596(7871):257–61. 34349261 10.1038/s41586-021-03778-8 PMC8756380

[18] Brittin CA, Cook SJ, Hall DH, Emmons SW, Cohen N. A multi-scale brain map derived from whole-brain volumetric reconstructions. Nature. 2021;591(7848):105–10. 33627874 10.1038/s41586-021-03284-x PMC11648602

[19] White JG, Southgate E, Thomson JN, Brenner S. Factors that determine connectivity in the nervous system of Caenorhabditis elegans. Cold Spring Harb Symp Quant Biol. 1983Jan;1(48):633–40. 10.1101/sqb.1983.048.01.067 6586380

[20] Moyle MW, Barnes KM, Kuchroo M, Gonopolskiy A, Duncan LH, Sengupta T, et al. Structural and developmental principles of neuropil assembly in C. elegans. Nature. 2021;591(7848):99–104. 33627875 10.1038/s41586-020-03169-5 PMC8385650

[21] Steuer Costa W, Van der Auwera P, Glock C, Liewald JF, Bach M, Schüler C, et al. A GABAergic and peptidergic sleep neuron as a locomotion stop neuron with compartmentalized Ca2+ dynamics. Nat Commun. 2019;10(1):4095. 10.1038/s41467-019-12098-5 PMC6736843 31506439

[22] Ouellette MH, Desrochers MJ, Gheta I, Ramos R, Hendricks M. A gate-and-switch model for head orientation behaviors in Caenorhabditis elegans. eNeuro. 2018 [cited 2024 July 27];5(6). Available from: https://www.eneuro.org/content/5/6/ENEURO.0121-18.2018 10.1523/ENEURO.0121-18.2018 PMC6325537 30627635

[23] Hendricks M, Ha H, Maffey N, Zhang Y. Compartmentalized calcium dynamics in a C. elegans interneuron encode head movement. Nature. 2012;487(7405):99–103. 10.1038/nature11081 PMC3393794 22722842

[24] Tao L, Porto D, Li Z, Fechner S, Lee SA, Goodman MB, et al. Parallel processing of two mechanosensory modalities by a single neuron in C. elegans. Dev Cell. 2019;51(5):617–631.e3. 10.1016/j.devcel.2019.10.008 PMC8635448 31735664

[25] Liu H, Yang W, Wu T, Duan F, Soucy E, Jin X, et al. Cholinergic Sensorimotor Integration Regulates Olfactory Steering. Neuron. 2018Jan 17;97(2):390-405.e3. 29290549 10.1016/j.neuron.2017.12.003 PMC5773357

[26] Hendricks M, Zhang Y. Complex RIA calcium dynamics and its function in navigational behavior. Worm. 2013July 1;2(3):e25546. 24778936 10.4161/worm.25546 PMC3875648

[27] Douglas RJ, Koch C, Mahowald M, Martin KA, Suarez HH. Recurrent excitation in neocortical circuits. Science. 1995Aug 18;269(5226):981–5. 7638624 10.1126/science.7638624

[28] Bogacz R, Brown E, Moehlis J, Holmes P, Cohen JD. The physics of optimal decision making: a formal analysis of models of performance in two-alternative forced-choice tasks. Psychol Rev. 2006;113(4):700–65. 17014301 10.1037/0033-295X.113.4.700

[29] Azulay A, Itskovits E, Zaslaver A. The C. elegans connectome consists of homogenous circuits with defined functional roles. PLoS Comput Biol. 2016;12(9):e1005021. 10.1371/journal.pcbi.1005021 PMC5015834 27606684

[30] Kashtan N, Itzkovitz S, Milo R, Alon U. Topological generalizations of network motifs. Phys Rev E. 2004;70(3):031909. 10.1103/PhysRevE.70.031909 15524551

[31] White J. Clues to basis of exploratory behaviour of the C. elegans snout from head somatotropy. Philos Trans R Soc B Biol Sci. 2018;373(1758):20170367. 10.1098/rstb.2017.0367 PMC6158226 30201833

[32] Peters A, Feldman ML. The projection of the lateral geniculate nucleus to area 17 of the rat cerebral cortex. I General description J Neurocytol. 1976Feb 1;5(1):63–84. 1249593 10.1007/BF01176183

[33] Rees CL, Moradi K, Ascoli GA. Weighing the evidence in Peters’ rule: does neuronal morphology predict connectivity? Trends Neurosci. 2017;40(2):63–71. 28041634 10.1016/j.tins.2016.11.007 PMC5285450

[34] Cook SJ, Kalinski CA, Hobert O. Neuronal contact predicts connectivity in the *C. elegans* brain. Curr Biol. 2023;33(11):2315–2320.e2. 10.1016/j.cub.2023.04.071 37236179

[35] Sanes JR, Zipursky SL. Synaptic specificity, recognition molecules, and assembly of neural circuits. Cell. 2020Apr 30;181(3):536–56. 32359437 10.1016/j.cell.2020.04.008

[36] Valdes-Aleman J, Fetter RD, Sales EC, Heckman EL, Venkatasubramanian L, Doe CQ, et al. Comparative connectomics reveals how partner identity, location, and activity specify synaptic connectivity in Drosophila. Neuron. 2021Jan 6;109(1):105-122.e7. 33120017 10.1016/j.neuron.2020.10.004 PMC7837116

[37] Milo R, Shen-Orr S, Itzkovitz S, Kashtan N, Chklovskii D, Alon U. Network Motifs: Simple Building Blocks of Complex Networks. Science. 2002Oct 25;298(5594):824–7. 12399590 10.1126/science.298.5594.824

[38] Reigl M, Alon U, Chklovskii DB. Search for computational modules in the C. elegansbrain. BMC Biol. 2004;2(1):25. 10.1186/1741-7007-2-25 PMC539283 15574204

[39] Varshney LR, Chen BL, Paniagua E, Hall DH, Chklovskii DB. Structural properties of the Caenorhabditis elegans neuronal network. PLOS Comput Biol. 2011Feb 3;7(2):e1001066. 21304930 10.1371/journal.pcbi.1001066 PMC3033362

[40] Itskovits E, Ruach R, Kazakov A, Zaslaver A. Concerted pulsatile and graded neural dynamics enables efficient chemotaxis in C. elegans. Nat Commun. 2018;9(1):2866. 10.1038/s41467-018-05151-2 PMC6054637 30030432

[41] Pritz C, Itskovits E, Bokman E, Ruach R, Gritsenko V, Nelken T, et al. Principles for coding associative memories in a compact neural network. Iino Y, Behrens TE, editors. eLife. 2023;12:e74434. 10.7554/eLife.74434 PMC10159626 37140557

[42] Towlson EK, Vértes PE, Ahnert SE, Schafer WR, Bullmore ET. The rich club of the C. elegans neuronal connectome. J Neurosci Off J Soc Neurosci. 2013;33(15):6380–7. 10.1523/JNEUROSCI.3784-12.2013 PMC4104292 23575836

[43] Sabrin KM, Wei Y, van den Heuvel MP, Dovrolis C. The hourglass organization of the Caenorhabditis elegans connectome. PLOS Comput Biol. 2020Feb 6;16(2):e1007526. 32027645 10.1371/journal.pcbi.1007526 PMC7029875

