# Supplementary material for: Emergence of clustered synapses during the development of a nervous system
Source: BMC Biol. 2026 Feb 5;24:65. doi: 10.1186/s12915-026-02539-1 (PMC12964600; doi:10.1186/s12915-026-02539-1)
Supplement: Supplementary file 1 — Additional file 1. Supplementary Figures S1–S8. A pdf file consisting of eight supplementary figures. Supplementary Fig. 1. Control analysis for synaptic clusters showing that the chosen neurite proximity does not affect the cluster dynamics throughout development. Supplementary Fig. 2. The number of synapses forming clustered connections increases between larval and adult stages. Supplementary Fig. 3. Number and fraction of postsynaptic neurons and muscles which are connected by clustered synapses to a presynaptic motor neuron. Supplementary Fig. 4. Emergence of tri-neuron circuits during development. Supplementary Fig. 5. The RIA interneuron emerges as the primary interneuron within the overrepresented tri-neuron circuits to form clustered synapses. Supplementary Fig. 6. Comparison of neuron pairs with clustered synapses between datasets. Supplementary Fig. 7. Similarity of neuron pairs connected by at least three synapses. Supplementary Fig. 8. Similarity of neuron pairs connected by at least four synapses. [file 12915_2026_2539_MOESM1_ESM.pdf]

## Additional file 1. Supplementary figures

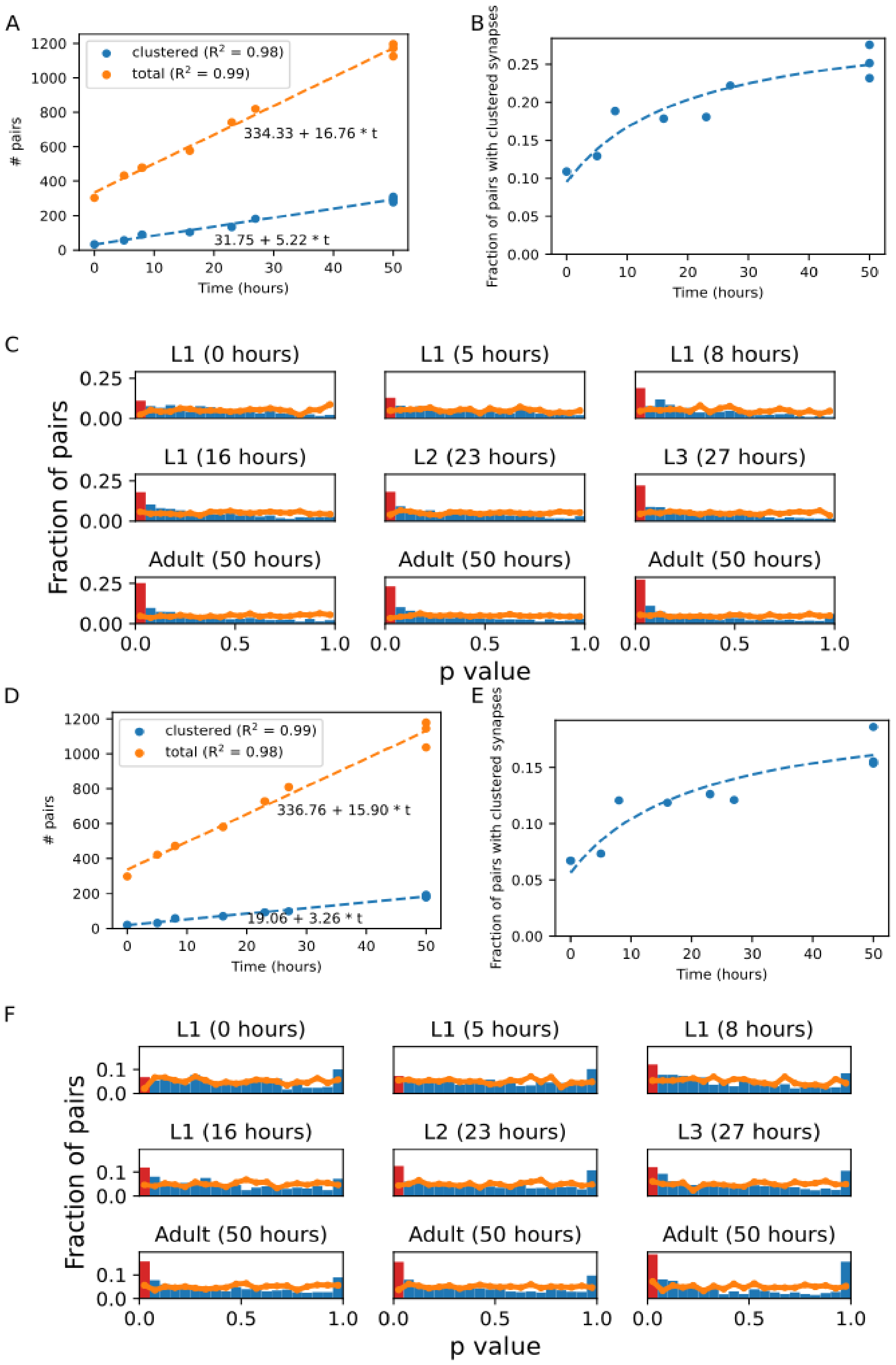

**Supplementary Figure 1. Control analysis for synaptic clusters showing that the chosen neurite proximity does not affect the cluster dynamics throughout development.**

Here we assumed neurite proximity 20% larger (A-C) or 20% lower (D-F) than the one considered throughout the study and for which a similar analysis is shown in figure 1.

(**A, D**) Number of connected pairs of neurons. Orange: total number of connected pairs. Blue: number of connected pairs in which the synapses are clustered.

(**B, E**) Fraction of connected pairs of neurons with clustered synapses out of the total number of connected pairs (trend lines are calculated from the linear fits in (A) and (D) respectively).

(**C, F**) Distributions of p-values of synapse clustering between neuron pairs in each dataset obtained from permutation tests (see Methods). Orange line shows the p-value distribution of the null-model where clustering of randomly positioned synapses is tested. Real synapse positions show significantly enriched clustering (red bar) compared to the null model (Kolmogorov-Smirnov test, $p<0.001$ for all datasets in (C) and $p<0.005$ for all datasets except for 0 and 5 hours in (F)).

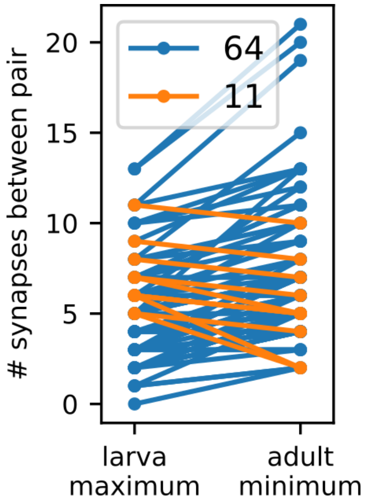


**Supplementary Figure 2. The number of synapses forming clustered connections increases between larval and adult stages.**

Each line connects the maximal number of synapses in the larval stages with the minimal number of synapses in the young adult stage. In blue are the cases in which the number of synapses in the adult stage is greater than or equal to the maximal number of synapses in the larval stages, and in orange are the cases where there are fewer synapses in the adult than there were in the larval stages.

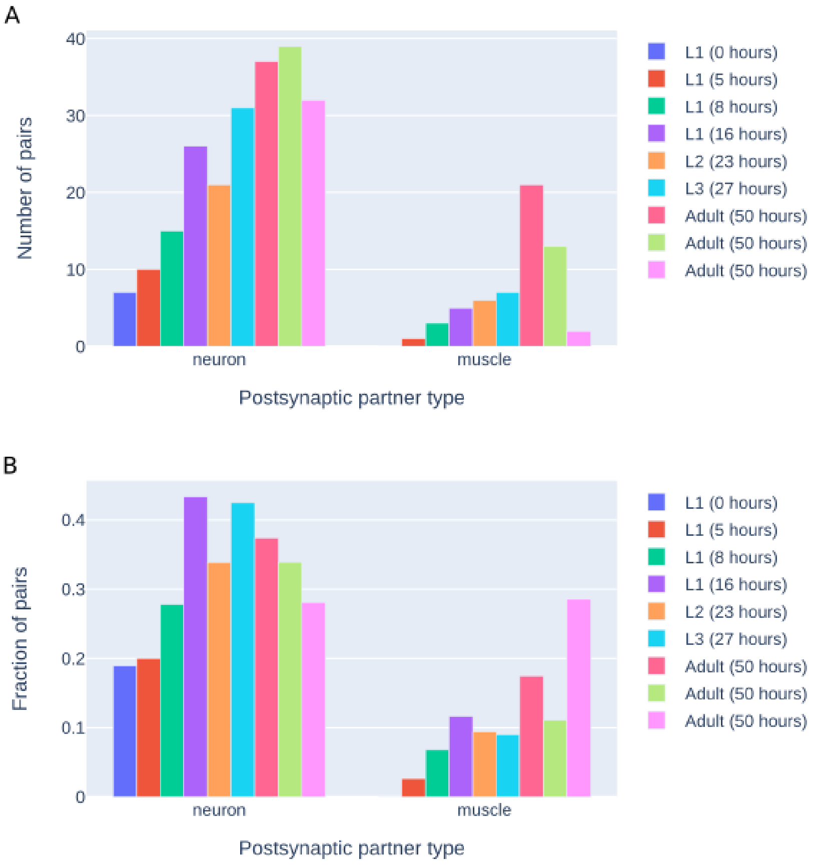


**Supplementary Figure 3. Number and fraction of postsynaptic neurons and muscles which are connected by clustered synapses to a presynaptic motor neuron.**

- **(A)** The number of postsynaptic neurons and muscles that are connected by clustered synapses to a presynaptic motor neuron. Most of the clustered synapse connections formed by a presynaptic motor neuron are with other neurons and not with muscles.
- **(B)** Fraction of connected presynaptic motor neurons and postsynaptic neurons or muscles that are connected by clustered synapses. Even when accounting for the fewer number of connections made by motor neurons to muscles than to other neurons, most of the clustered synapses are with other neurons rather than with muscles.

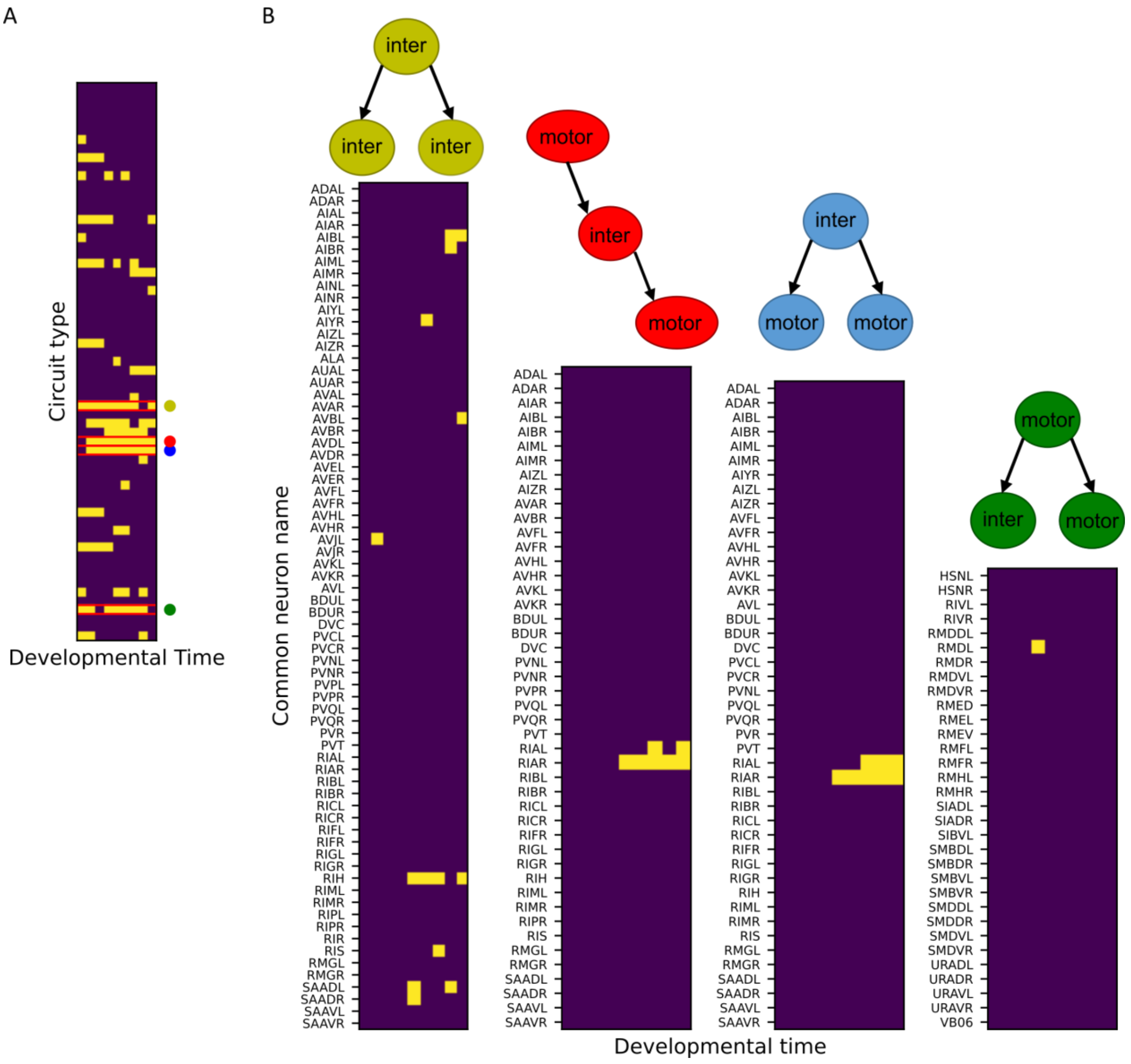


**Supplementary Figure 4. Emergence of tri-neuron circuits during development.**

**(A)** Heat map showing which circuit types are significantly clustered (yellow) in each dataset (permutation test). Four circuit types stood out, from top to bottom: a presynaptic interneuron which connects to two postsynaptic interneurons (yellow dot), an interneuron which is both pre- and postsynaptic to motor neurons (red dot), a presynaptic interneuron which connects to two postsynaptic motor neurons (blue dot), and a motor neuron which is presynaptic to both an interneuron and a motor neuron (green dot).

**(B)** Heat map showing which neurons are over represented as having clustered synapses in each of the overrepresented clustered circuit types (left to right: a presynaptic interneuron which connects to two postsynaptic interneurons (yellow dot), an interneuron which is both pre- and postsynaptic to motor neurons (red dot), a presynaptic interneuron which connects to two postsynaptic motor neurons (blue dot), and a motor neuron which is presynaptic to both an interneuron and a motor neuron (green dot)).

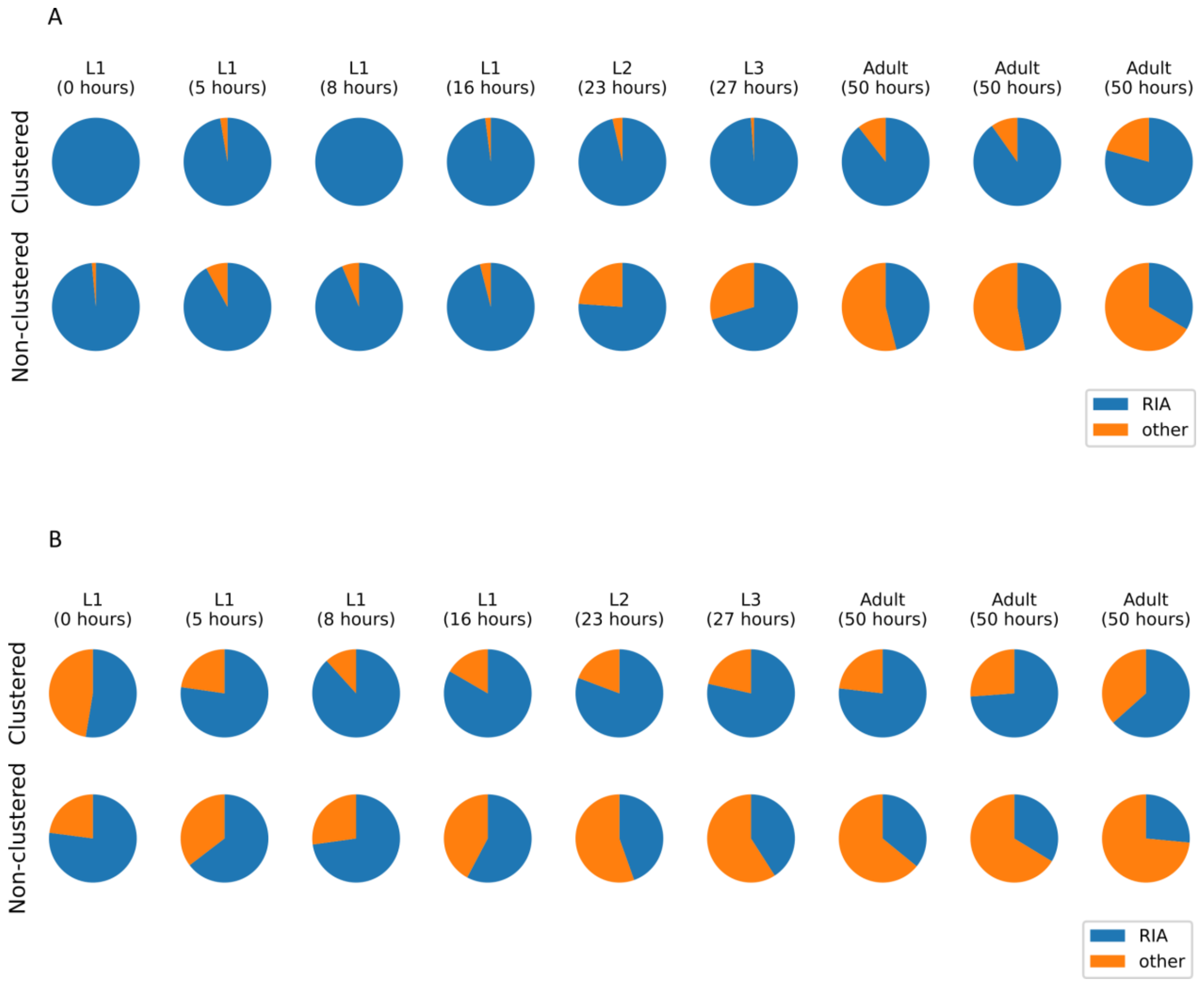


**Supplementary Figure 5. The RIA interneuron emerges as the primary interneuron within the overrepresented tri-neuron circuits to form clustered synapses.**

- **(A)** Fractions of the overrepresented linear circuits with clustered (top) and non-clustered (bottom) synapses with RIA as the shared connecting interneuron.
- **(B)** Fractions of the overrepresented mutually regulating circuits with clustered (top) and non-clustered (bottom) synapses with RIA as the shared pre-synaptic interneuron.

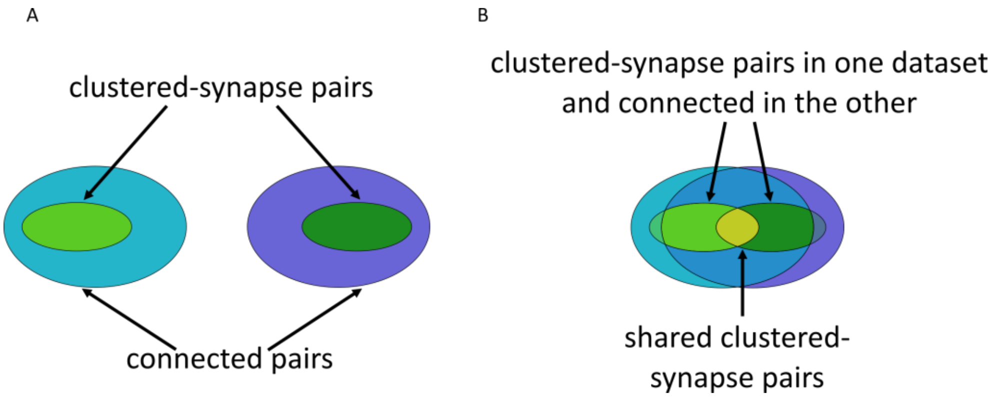


**Supplementary Figure 6. Comparison of neuron pairs with clustered synapses between datasets.**

**(A)** Two datasets of connected pair of neurons, each containing a subset of pairs connected by clustered synapses.

**(B)** The number of neuronal pairs with clustered synapses in both datasets (yellow) was divided by the number of pairs with clustered synapses that also appear in the other dataset (the two green sections).

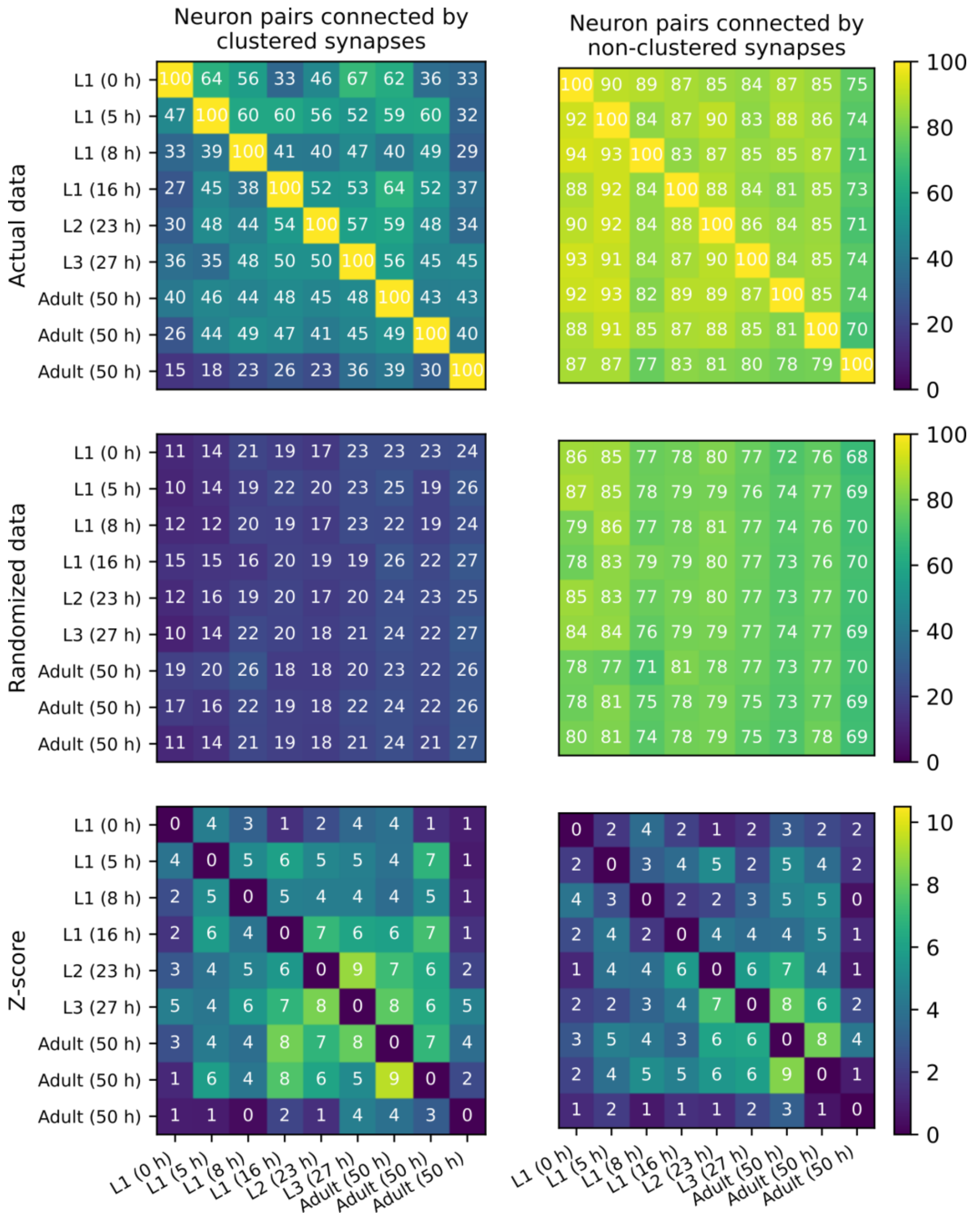


**Supplementary Figure 7. Similarity of neuron pairs connected by at least three synapses.**

**Top:** A pairwise analysis indicating the % of neural pairs with clustered synapses in one developmental stage (denoted by the row) that also have clustered synapses in the other developmental stage (denoted by the column). **Middle:** Repeating the analysis using bootstrap to correct for the number of neural pairs with clustered and non-clustered synapses (see

Methods). N=50 iterations. **Bottom:** Z-scores calculated based on the top and the middle matrices. Left and right panels denote the neural pairs connected by clustered and non-clustered synapses, respectively. The values of the right most and the bottom are for the adult animal connectome compiled by [16]. All developmental stages as well as the two other adult connectomes are from [17].

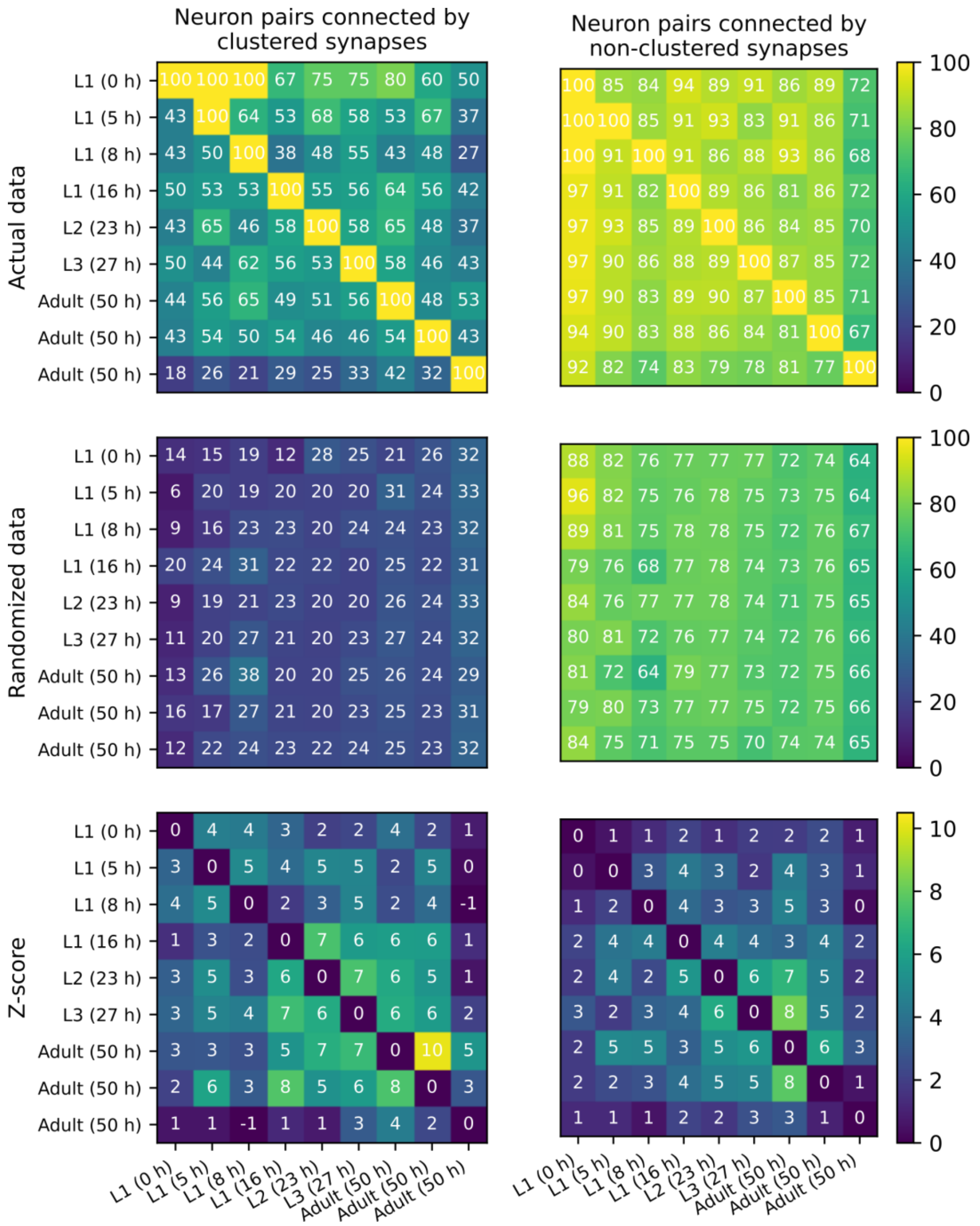


**Supplementary Figure 8. Similarity of neuron pairs connected by at least four synapses.**

**Top:** A pairwise analysis indicating the % of neural pairs with clustered synapses in one developmental stage (denoted by the row) that also have clustered synapses in the other developmental stage (denoted by the column). **Middle:** Repeating the analysis using bootstrap to correct for the number of neural pairs with clustered and non-clustered synapses (see

Methods). N=50 iterations. **Bottom:** Z-scores calculated based on the top and the middle matrices. Left and right panels denote the neural pairs connected by clustered and non-clustered synapses, respectively. The values of the right most and the bottom are for the adult animal connectome compiled by [16]. All developmental stages as well as the two other adult connectomes are from [17].
